# Prognostic Role of Lymphocyte-C-Reactive Protein Ratio in Colorectal Cancer: A Systematic Review and Meta Analysis

**DOI:** 10.3389/fonc.2022.905144

**Published:** 2022-07-06

**Authors:** Xinglong He, Ade Su, Yongcheng Xu, Diaolong Ma, Guoyuan Yang, Yiyun Peng, Jin Guo, Ming Hu, Yuntao Ma

**Affiliations:** ^1^The First Clinical School of Gansu University of Chinese Medicine, Lanzhou, China; ^2^General Surgery Clinical Medical Center, Gansu Provincial People’s Hospital, Lanzhou, China; ^3^Gansu Provincial Key Laboratory of Molecular Diagnosis and Precision Therapy of Surgical Tumors, Gansu Provincial People’s Hospital, Lanzhou, China; ^4^The Second Clinical Medical College of Lanzhou University, Lanzhou, China; ^5^The first Department of general surgery, Gansu Provincial People's Hospital, Lanzhou, China

**Keywords:** colorectal tumors, lymphocyte-C-reactive protein ratio, prognosis, systematic evaluation, metaanalysis

## Abstract

**Introduction:**

The lymphocyte-C-reactive protein ratio (LCR) is a new immunoinflammatory score and prognostic marker, but the relationship between this index and the prognosis of colorectal cancer patients remains controversial.Therefore, aim of the study was to assess the relationship between LCR and prognosis for colorectal cancer patients through a systematic evaluation and meta-analysis.

**Methods:**

We systematically searched PubMed, EMBASE, Web of Science, and Cochrane Library databases for randomized controlled studies and observational studies on the relationship between LCR and prognosis of colorectal cancer patients, all searched from the date of database creation to January 6, 2022.Our primary endpoints observed were overall survival (OS) and disease-free survival (DFS) of colorectal cancer patients, and secondary observables were basic characteristics of included studies, such as country, study duration, sample size, LCR threshold, and pathological characteristics of patients in each study, such as degree of differentiation, gender, tumor location, T stage, and lymphatic metastasis.

**Results:**

A total of 10 case-control studies including 7068 patients were included. Meta-analysis results showed that overall survival (OS) and disease-free survival (DFS) were worse in colorectal cancer patients with lower levels of LCR (HR=0.44, 95% CI=0.38-0.52, P<0.001; HR=0.56, 95% CI=0.41-0.76, P< 0.001).Subgroup analysis based on country, study length, sample size, and LCR threshold showed that lower levels of LCR were all associated with poorer OS (P < 0.05). Regarding pathological characteristics, patients in the low LCR group were generally poorly differentiated (OR=1.79, 95% CI=1.55-2.07, P<0.001), while there was no significant relationship with gender, tumor location, T stage, and lymphatic metastasis (P>0.05).

**Discussion/Conclusion:**

LCR can be used as a prognostic marker for colorectal cancer patients, and patients with lower levels of LCR may have a poor prognosis. Due to the limitation of the number and quality of the included studies, the above findings need to be validated by more high-quality studies.

**Systematic Review Registration:**

https://www.crd.york.ac.uk/prospero/, identifier CRD42022296563.

## Introduction

Colorectal cancer (CRC) is one of the leading causes of cancer deaths worldwide, with more than 1.9 million new colorectal cancer cases and 935,000 deaths estimated worldwide in 2020, accounting for approximately one tenth of cancer cases and deaths.Overall, colorectal cancer ranked third in terms of incidence and second in terms of mortality (1). Despite recent advances in multidisciplinary treatment, including surgery, chemotherapy and radiotherapy,however, mortality from CRC remains high, especially in patients with distant metastases or postoperative recurrence, even after curative surgery.Pathological TNM staging is currently the most effective prognostic indicator after surgery, but remains inadequate (2). The development of optimal biomarkers for predicting recurrence or poor prognosis is very important at this time in order to better formulate treatment plans for patients in the clinic.

Cancer-associated inflammation is considered to be one of the key components of tumors and may represent a seventh feature of cancer (3, 4). It is also a well-established paradigm that inflammation is closely associated with cancer development, including carcinogenesis and tumor progression (e.g., invasion, migration, and metastasis) (5). In contrast, postoperative complications aggravate the prognosis of patients with malignancy, increase the levels of inflammatory cytokines, such as interleukin-6 (IL-6), and may lead to the proliferation of residual cancer cells (6–12). On the other hand, c-reactive protein (CRP) is the most common indicator of systemic inflammation and is closely associated with serum IL-6 levels (13, 14). Studies have also shown that lymphocyte levels are an independent prognostic factor for certain cancer types, such as breast, colorectal and pancreatic cancers (15–17). In addition, lymphocyte subsets infiltrated by tumors, such as CD8+ T cells and memory T cells, are associated with better prognosis in various tumors (18, 19).

Since tumor patients have both inflammatory infections and immune disorders after surgery, and CRP and lymphocyte levels precisely also reflect well the inflammatory infections and immune status of patients after surgery, a series of studies on colorectal cancer patients in recent years have defined the ratio of lymphocytes to CRP as a new index and reported the correlation between LCR and prognosis of colorectal cancer (20–29). However, the findings of the studies are not uniform and the cut-off values for this index are not consistent; therefore, this study used Meta-analysis to objectively and systematically investigate the prognostic significance of treatment LCR levels in colorectal cancer patients and the relationship between LCR and the clinicopathological characteristics of colorectal cancer patients.

## Data and Methods

### Search Strategy

Computer searches of PubMed, EMBASE, Web of Science, Cochrane Library databases, and finding gray literature were conducted to collect published cohort studies on the relationship between LCR and colorectal cancer prognosis by January 2022. The search was performed using a combination of subject terms and free words. Moreover, references of the included articles were traced for relevant literature. The search terms included: Colorectal Neoplasms, Colorectal Neoplasm, Colorectal Tumors, Colorectal Tumor, Colorectal Cancer, Colorectal Carcinoma, Colorectal Carcinomas, lymphocyte c-reactive protein ratio, lymphocyte/c-reactive protein ratio, LCR. this study has been pre-registered in PROSPERO (registration number: CRD42022296563). Inclusion and exclusion criteria

Inclusion criteria (1) Study type: cohort study; (2) Study population: patients who have published studies exploring the relationship between LCR and prognosis of colorectal cancer patients and who have been pathologically diagnosed with colorectal cancer; (3) Outcome indicators: OS, DFS, recurrence-free survival (RFS).

Exclusion criteria (1) The type of article is a review, systematic evaluation, conference paper, expert review; (2) The full text of the literature is not available; (3) Insufficient data; (4) Duplicate published literature; (5) Non-English literature.

Literature screening and data extraction were performed by 2 researchers who independently screened the literature, extracted the data and cross-checked them, if a dispute arose it was resolved through a third researcher. The literature is screened by first reading the title and abstract, and after excluding the obviously irrelevant literature, read the full text further to determine whether to include it. If needed, the original study authors were contacted by email or phone for information not identified but important to this study. Data extraction includes: study title, first author, year of publication, study duration, country, sample size, gender, treatment modality, LCR threshold, threshold cut-off method, outcome indicators of interest, and pathological characteristics. The data required for the ending metrics were extracted from the literature survival curves by Engauge Digitizer software.

The quality of the literature was evaluated using the Cochrane Risk of Bias tool to evaluate the quality of randomized controlled trials and the Newcastle-Ottawa Scale for observational studies, and studies with a score of 6 or higher were defined as high quality studies (30).

### Statistical Processing

Stata 12.0 software was used to analyze the data. The relationship between LCR and prognosis of colorectal cancer patients was evaluated by HR and its 95% CI. The relationship between LCR and OS and DFS was explored separately. To further investigate the effect of LCR on the prognosis of colorectal cancer patients, subgroup analysis was performed on the LCR threshold, country, sample size, and study duration. Heterogeneity of the included literature was determined by the I2 statistic and q test. Heterogeneity was significant when P<0.1 and/or I2>50%, and Meta-analysis was performed using a random-effects model; conversely, Meta-analysis was performed using a fixed-effects model (31). Begg’s test and Egger’s test were used to test for potential publication bias (test level α=0.05) (32).

## Results

**Figure 1:**
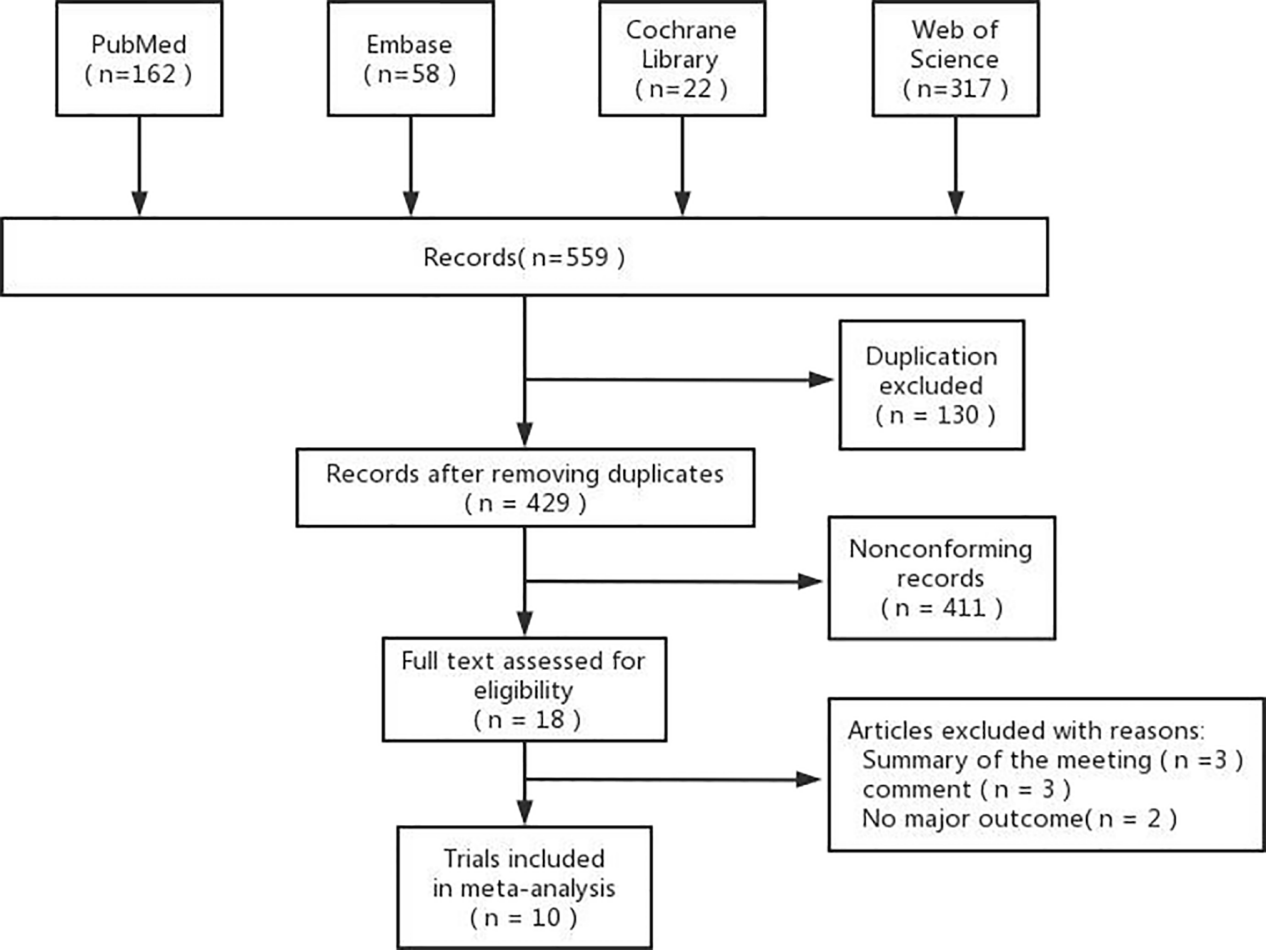
Flowchart of study inclusion.

Through our initial search strategy, we identified a total of 559 records, obtained 429 documents after weighting, excluded 429 documents that did not meet the inclusion criteria, initially included 18 relevant documents, further reading of the full text excluded 3 conference abstracts, 3 reviews, and 2 without primary outcome indicators, and finally included 10 retrospective cohort studies (**Figure 1**).

### Basic Characteristics of the Included Studies

A total of 10 retrospective cohort studies including 7068 patients were included. The included studies were published in 2019-2021, 8 conducted in Japan and 2 in China.All 10 studies reported the relationship between LCR and OS, and 5 studies reported the relationship between LCR and DFS. Eight of them defined LCR as lymphocytes/C-reactive protein ratio, while the other two used C-reactive protein/lymphocytes ratio (CLR) (24, 29).The study by Taniai et al. could be used for OS and DFS analysis after data extraction, while the study by Yongsheng Meng et al. could not extract OS outcome data but could be used for analysis of pathological characteristics factors and was therefore included. The LCR threshold values ranged from 5000 to 15923. The NOS scores of the included studies were all above 6, suggesting a high quality of the included studies (**Table 1**).

**Table 1:** Characteristics of included studies.

The included studies	Country	Research time	Cohort/n	Male/female(n)	End index	LCR determined WAY	LCR critical value	Surgical operation	TNM Stage	NOS
Okugawa, et al., 2019 (20)	Japan	2004- 2011	377	285/192	OS/DFS	ROC analysis	6000	surgery	I-IV	6
Suzuki, et al 2019 (28)	Japan	2004- 2013	1303	689/614	OS/DFS	ROC analysis	12980	surgery + chemo	I-IV	7
Nakamura, et al., 2021 (26)	Japan	2000-2015	756	435/321	OS	ROC analysis	5000	chemo	unresectable	7
Nishi, et al., 2021 (22)	Japan	2004- 2012	48	32/16	OS/DFS	median	11765	surgery + chemo	I-III	8
Okugawa, et al., 2021 (23)	Japan	2006- 2015	307	183/124	OS/DFS	ROC analysis	6676	surgery	I-IV	7
Wenting Ou, et al., 2021 (25)	China	2010- 2014	955	540/415	OS	ROC analysis	6500	surgery + chemo	I-IV	6
Taniai, et al., 2020 (24)	Japan	2000- 2018	197	137/60	OS/DFS	ROC analysis	15923	surgery + chemo	Liver Metastases	6
Yasui, et al., 2020 (27)	Japan	2008- 2014	568	259/309	OS/RFS	ROC analysis	10424	surgery + chemo	III	8
Okugawa, et al., 2020 (21)	Japan	2001- 2015	86	64/22	OS/RFS	ROC analysis	6000	surgery + chemo	I-IV	8
Yongsheng Meng, et al., 2021 (29)	China	2004- 2019	2471	971/1500	OS	Maximally selected rank statistics	0.2	surgery + chemo	I-IV	6

### Meta-Analysis Results

#### The Relationship Between LCR and OS

**Figure 2:**
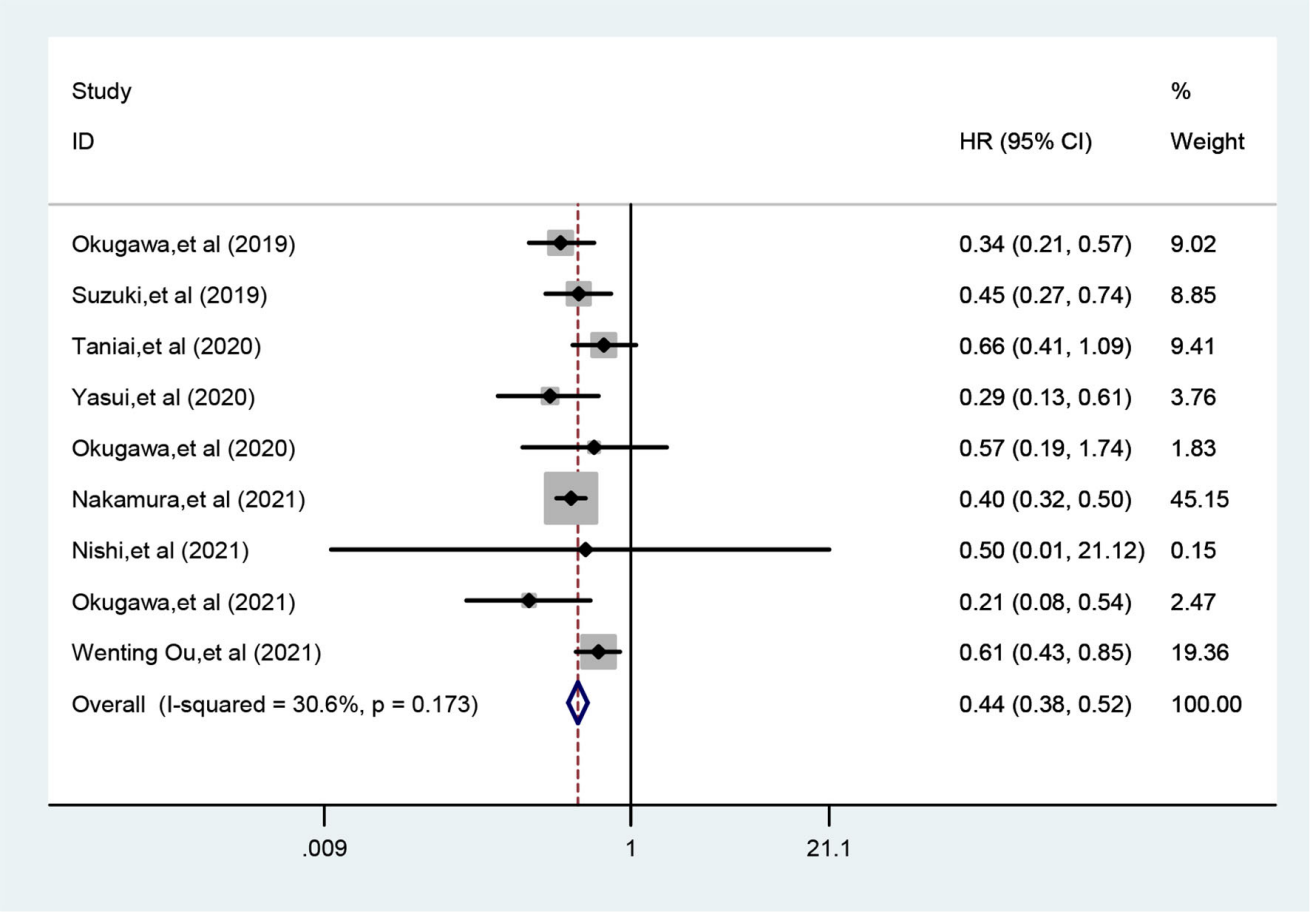
Meta-analysis of relationship between LCR and OS.

Nine studies reported the relationship between LCR and OS with no significant heterogeneity between studies (*I*^2 =^ 30.6%, *P*_h_=0.173), so a fixed-effects model was used. the results of the Meta-analysis showed that patients with lower LCR had significantly worse OS (HR=0.44, 95% CI=0.38-0.52, *P*<0.001) (**Figure 2**).

#### Relationship Between LCR and DFS

**Figure 3:**
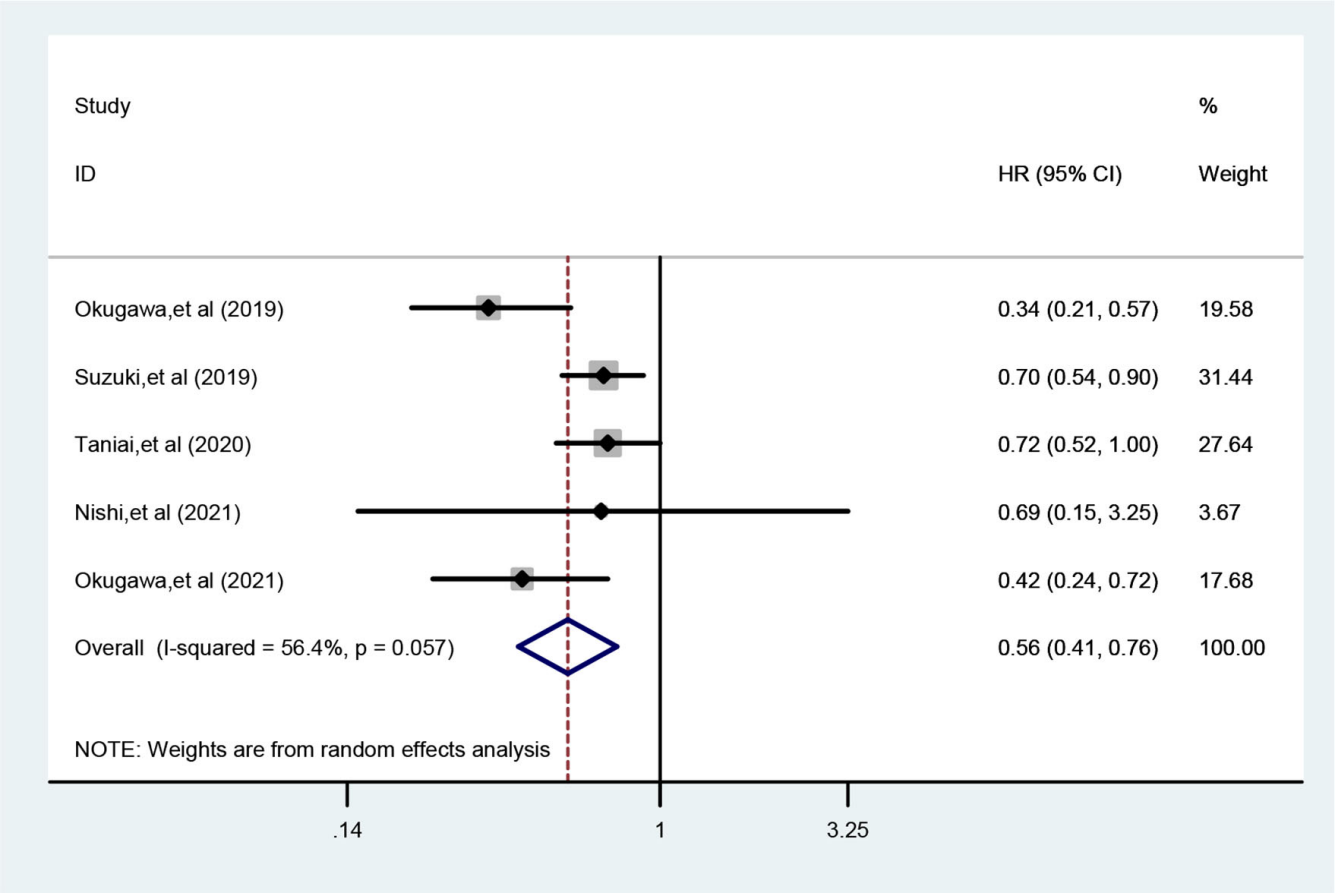
Meta-analysis of relationship between LCR and DFS.

Five studies reported the relationship between LCR and DFS with significant heterogeneity between studies (*I*^2 =^ 56.4%, *P*_h_=0.057), so a random-effects model was used. the results of Meta-analysis showed that patients with higher LCR had significantly shorter DFS/RFS (HR=0.56, 95% CI=0.41-0.76, *P*<0.001) (**Figure 3**).

### Subgroup Analysis 

To further investigate the prognostic value of LCR for patients with colorectal cancer, this study conducted a subgroup analysis in terms of country, study duration, sample size, and LCR threshold. The results showed that lower levels of LCR were all associated with poorer OS (*P* < 0.05) (**Table 2**).

**Table 2:** Subgroup analysis of relationship between LCR and OS.

Subgroup analysis	Number of studies (n)	Sample size (n)	Model	HR (95%CI)	P	Heterogeneity	
						I^2^(%)	P_h_
Country							
japan	8	3642	Fixed effect model	0.41 (0.34,0.49)	<0.001	5.0	0.329
china	1	955	Fixed effect model	0.61 (0.43,0.86)	0.004		
LCR critical value							
<10000	4	1725	Random effect model	0.42 (0.26,0.68)	<0.001	55.2	0.082
>10000	5	2872	Fixed effect model	0.43 (0.36,0.51)	<0.001	8.8	0.356
Research time							
<10 years	4	1948	Fixed effect model	0.47 (0.36,0.62)	<0.001	44.0	0.147
≥10 years	5	3649	Fixed effect model	0.43 (0.36,0.52)	<0.001	31.0	0.125
Sample size							
<400	5	1015	Fixed effect model	0.44 (0.32,0.61)	<0.001	35.3	0.186
≥400	4	3582	Fixed effect model	0.44 (0.37,0.51)	<0.001	43.9	0.148

### Relationship Between LCR and Clinicopathological Characteristics of Colorectal Cancer Patients

To investigate the relationship between LCR and clinicopathological characteristics of colorectal cancer patients, analysis was performed according to gender, tumor location, T stage, lymphatic metastasis, and degree of tumor differentiation. As shown in **Table 3**, patients in the low LCR group generally had a poorer degree of differentiation (OR=1.79, 95% CI=1.55-2.07, *P*<0.001), while there was no significant relationship with gender, tumor location, T stage, and lymphatic metastasis (*P*>0.05).

**Table 3:** Relations of LCR with clinicopathologic characteristics in patients with colorectal cancer.

Characteristic	Number of studies(n)	Sample size(n)	Model	OR(95%CI)	P	Heterogeneity	
						I^2^(%)	P_h_
Sex (male vs. female)	7	5816	Random effect model	0.96 (0.71,1.31)	0.799	78.4	< 0.001
Tumor location (right vs. left)	4	4727	Random effect model	1.49 (0.87,2.53)	0.144	91.9	< 0.001
T stage (I~II vs. III~IV)	4	4863	Random effect model	2.21 (0.93,5.21)	0.071	96.2	< 0.001
Lymphatic metastasis (yes vs. No)	4	4815	Random effect model	0.59 (0.27,1.28)	0.180	96.4	< 0.001
Degree of differentiation (well vs. Poor)	6	5619	Fixed effect model	1.79 (1.55,2.07)	< 0.001	48.6	0.083

### Sensitivity Analysis

**Figure 4:**
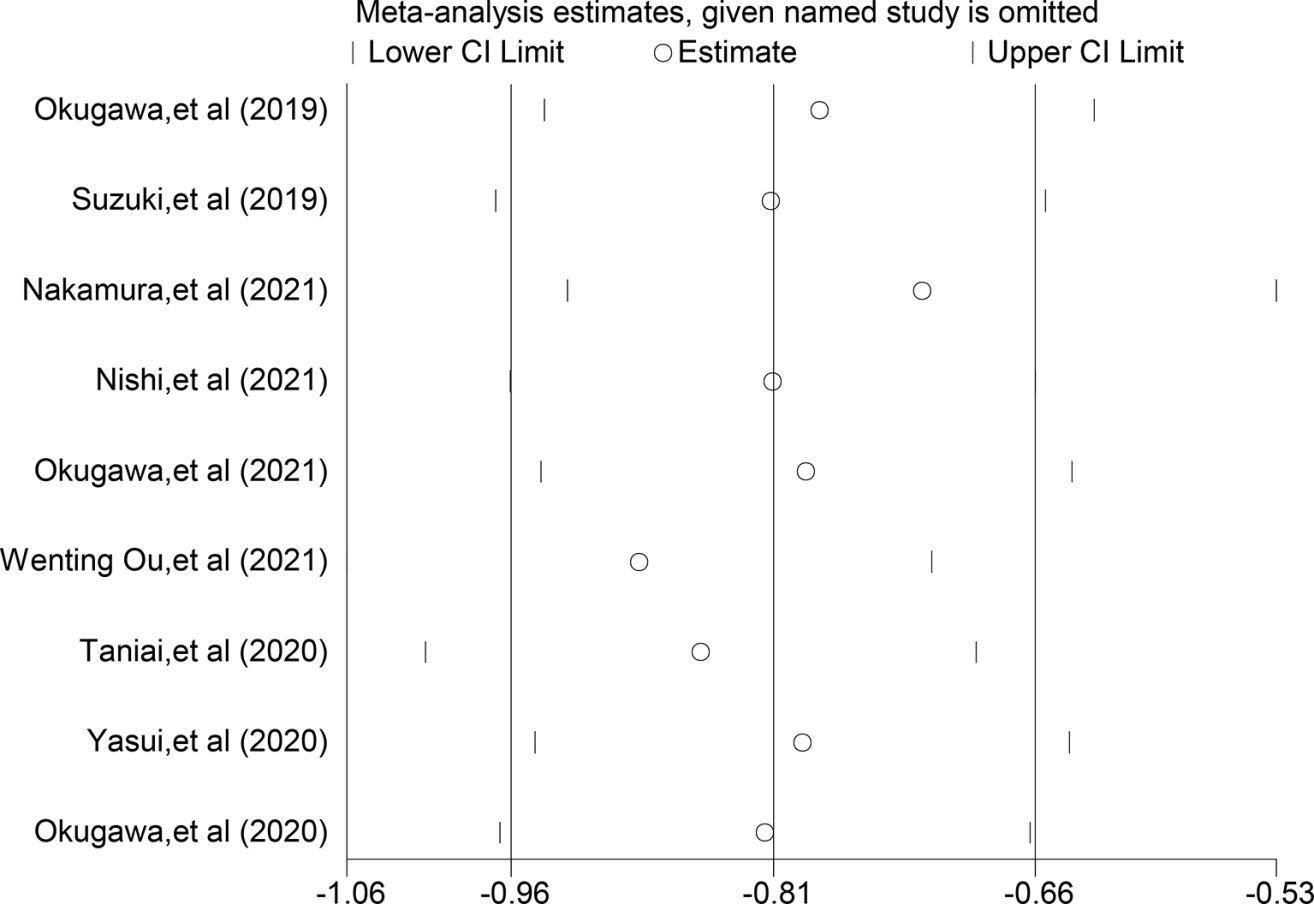
Sensitivity analysis of relationship between LCR and OS.

**Figure 5:**
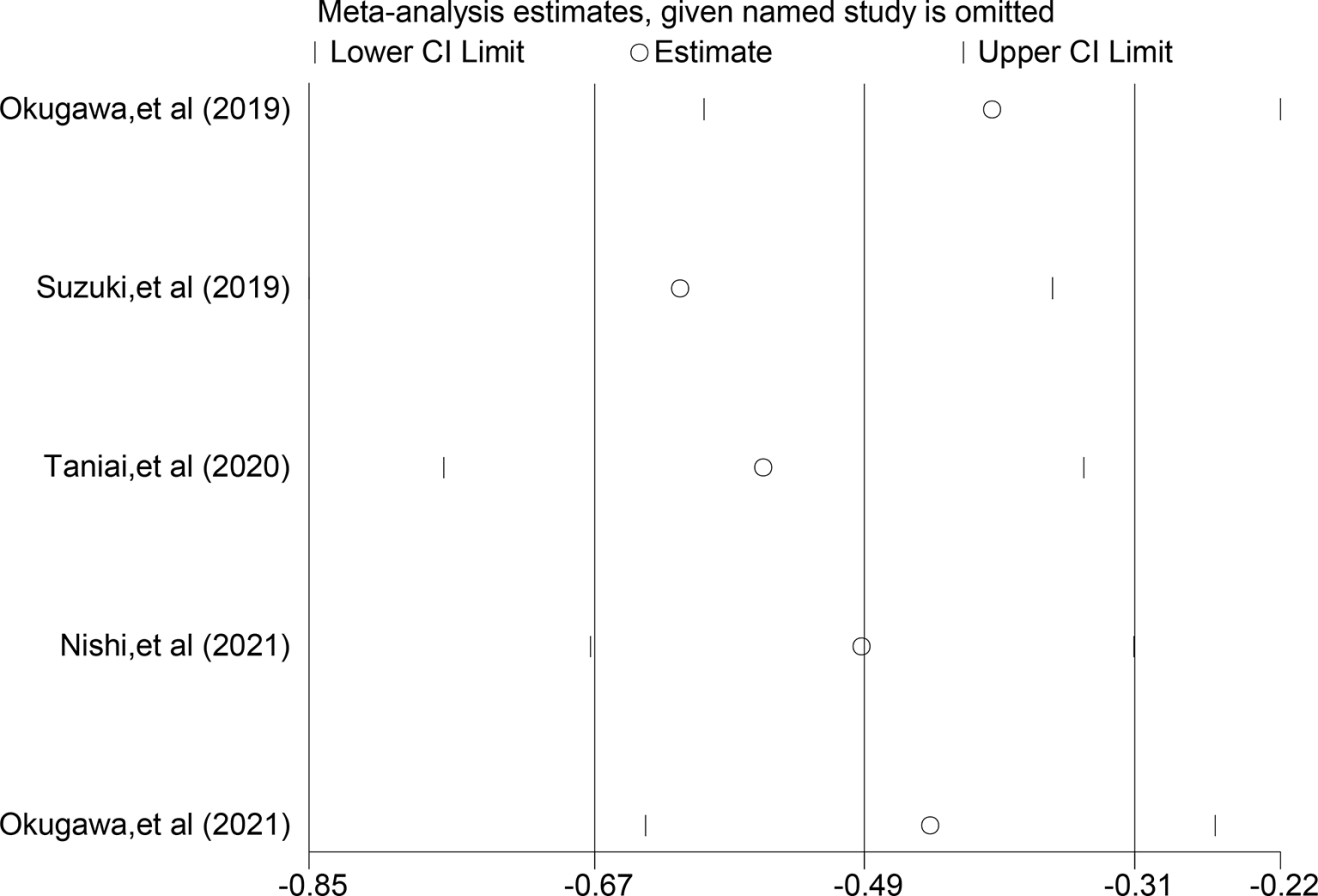
Sensitivity analysis of relationship between LCR and DFS.

Sensitivity analysis was performed by excluding individual studies one by one, and the results showed stable results for the Meta-analysis of the relationship between LCR and OS (**Figure 4**) and stable results for the Meta-analysis of the relationship between LCR and DFS (**Figure 5**).

## Publication Bias

**Figure 6:**
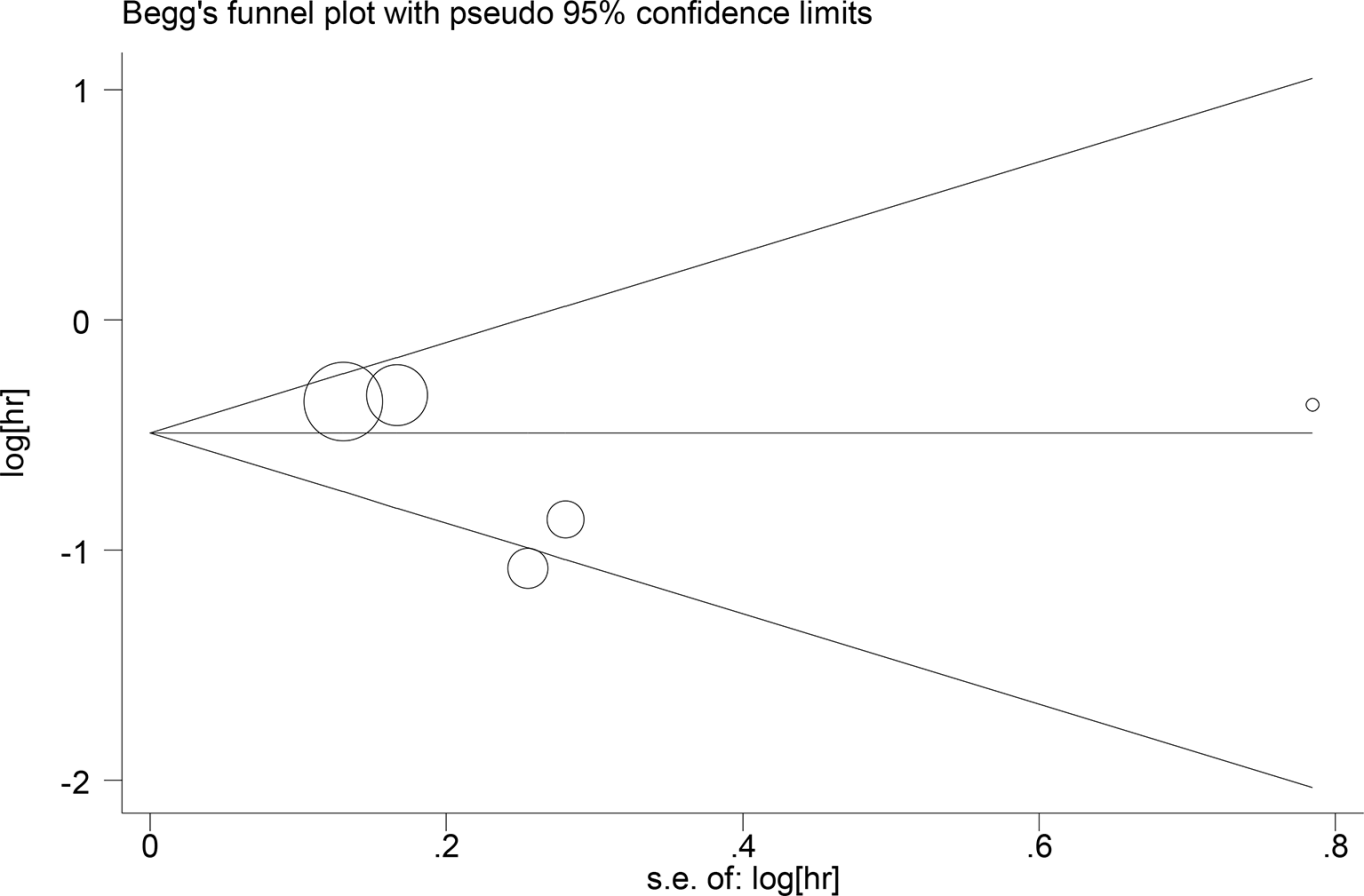
Begg’s test Relationship between LCR and OS.

**Figure 7:**
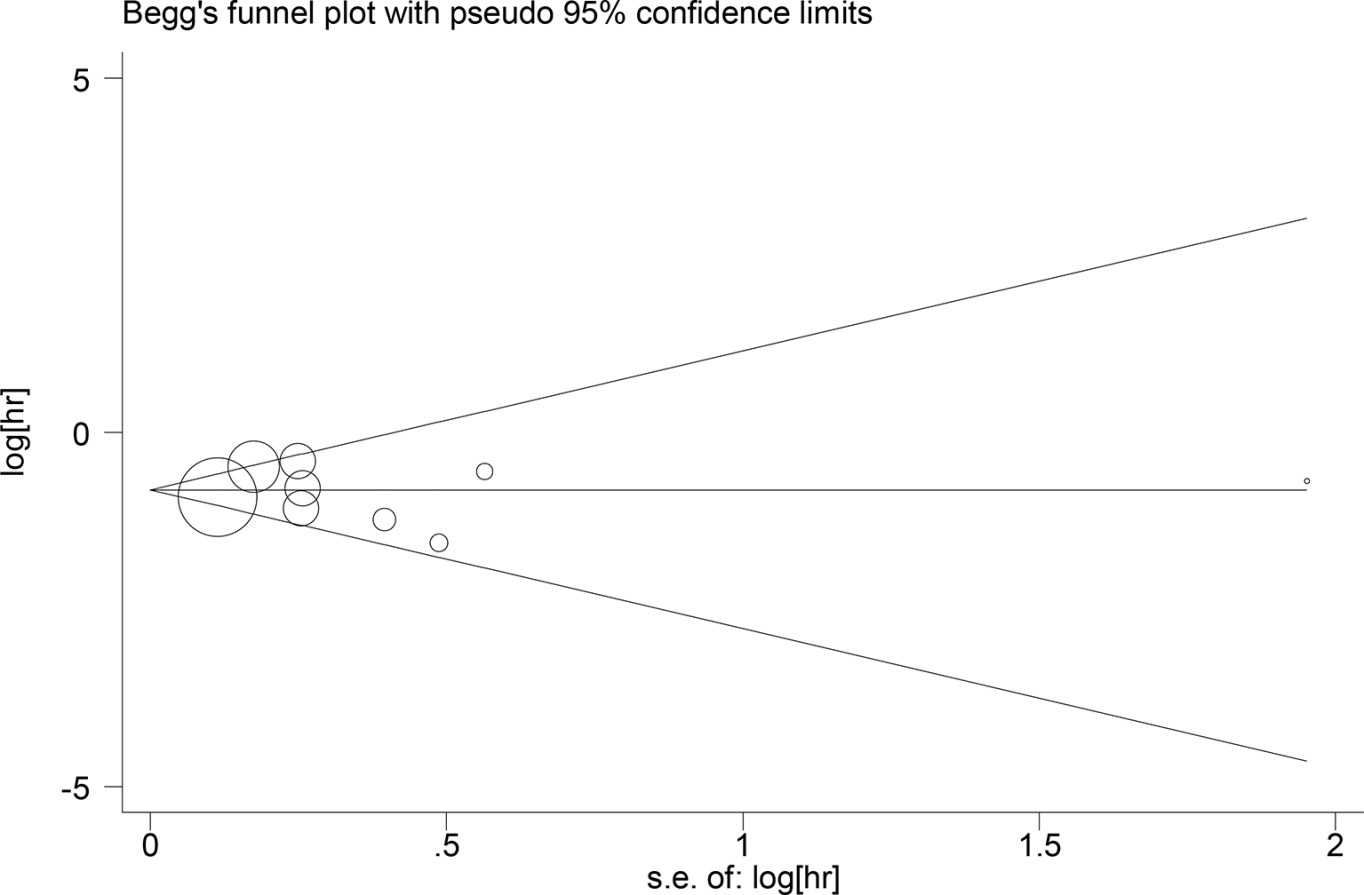
Begg’s test Relationship between LCR and DFS.

Publication bias for the relationship between LCR and OS was assessed by Begg’s test (Z=0.31, P=0.754), Egger’s test (t=-0.28, P=0.784) (**Figure 6**); publication bias for the relationship between LCR and DFS, Begg’s test (Z=0.73, P=0.462), Egger test (t=-1.02, P=0.381), the results indicated that the likelihood of publication bias in the included literature was low (**Figure 7**).

## Discussions

Many recent studies have shown that low LCR is associated with poor survival in patients with colorectal cancer. Here we performed a meta-analysis of 10 studies including 7068 colorectal cancer patients to assess the prognostic effect of LCR. The results showed a correlation between lower levels of LCR and shorter OS/DFS in colorectal cancer patients.

In recent years there has been increasing interest in the tumor microenvironment, where lymphocytes play a key role in the host immune response to cancer cells, while myeloid cells, such as neutrophils and macrophages, also appear to promote tumor growth by suppressing T-cell immunity through antagonistic cytokine signaling in the tumor microenvironment, such as interleukin (IL)-6 and transforming growth factor-b (33, 34).In the body circulation, CRP is associated with pro-tumorigenic effects, whereas IL-6 is usually produced by macrophages and is a regulator of CRP (35).Thus the amount of balance between lymphocytes and CRP can effectively reflect the dynamics of host-cancer interactions. Lymphocytes inhibit tumor cell proliferation and metastasis by promoting an immune response to the tumor and high CRP levels reflect proliferation and metastasis of tumor cells in patients with advanced cancer, with interleukin levels that can lead to cachexia and malnutrition, resulting in slower clearance of anticancer drugs and increased toxicity associated with therapy (36). On the other hand, relevant studies following the 2019 COVID-19 outbreak suggest that the LCR is potentially a predictive marker for COVID-19 infection and clinical deterioration, and given this evidence, the LCR may also reflect a frail or compromised host state (37–40).Therefore, increasing LCR may be an effective strategy to improve tumor prognosis in patients with unresectable metastatic colorectal cancer (26).

Okugawa (20) et al. showed that two peripheral blood parameters, lymphocyte count and C-reactive protein level, had the highest correlation with recurrence compared to other parameters, and low preoperative LCR was significantly associated with undifferentiated histology, advanced T-stage, lymph node metastasis, distant metastasis, and advanced grade; reduced preoperative LCR was an independent prognostic factor for disease-free and overall survival, and was an independent risk factor for postoperative complications and surgical site infections in colorectal cancer patients; Wenting Ou (25) et al. showed that LCR had the highest prognostic predictive value of all inflammatory scores and that low LCR was significantly associated with multiple clinicopathological features of tumor infiltration and progression. Nakamura (26) et al. showed in a study of patients with unresectable metastatic colorectal cancer that the lymphocyte-c-reactive protein ratio was the most sensitive predictor of sustained survival among all prognostic scores based on inflammation.

As a new prognostic marker, LCR reflects the prognostic status of cancer patients from a combined immune and inflammatory perspective. It is non-invasive and easier to detect, inexpensive, can be repeatedly sampled, and can achieve real-time tracking of tumor status, which makes it a hot spot in the field of emerging non-invasive tumor markers. The present meta-analysis showed that low level of LCR predicted worse prognosis in colorectal cancer patients, which is consistent with the findings of the included literature in this study, but the subgroup analysis showed no significant correlation between LCR and baseline characteristics and pathological features.This reflects many limitations of the current application of this index: all the studies were conducted in Asia and the inclusion of patients was too limited, future studies from other regions are needed to supplement them; The definition and threshold values of LCR were not sufficiently uniform across studies, with variations regarding the time points at which hematological samples were collected, yielding less reliable evidence; The sample sizes of some of the studies were too small and only retrospective studies were included, larger-sample and multicenter RCTs are needed; additionally, although the META analysis showed that low levels of LCR were associated with poorer OS and DFS, patients in the low LCR group generally had poorer differentiation and thus a poorer prognosis. Certainly, follow-up studies are needed to evaluate this bias, obtaining more valid evidences.

## Conclusion

LCR can be used as an auxiliary reference indicator for prognosis of colorectal cancer, and low LCR is associated with poor survival. LCR can be used as a cost-effective prognostic biomarker and as an assessment indicator in treatment decision making.

## Data Availability Statement

The original contributions presented in the study are included in the article/**Supplementary Material**. Further inquiries can be directed to the corresponding author.

## Author Contributions

XH: writing—original draft, formal analysis, conceptualisation, methodology, investigations, resources, writing—review and editing; AS: formal: analysis, conceptualisation, methodology, investigations, language polish; YX, DM, GY, YP, JG, MH contributed equally to this work: conceptualisation, writing—review and editing. YM: overall guidance, modification guide. All authors approved the final manuscript.

## Funding

Open Fund Project of Laboratory of Molecular Diagnosis and Precision Therapy of Surgical Tumors in Gansu Province (No. 2019GSZLSYS06). Gansu Provincial Hospital Scientific Research Fund Project (21GSSYB-6).

## Conflict of Interest

The authors declare that the research was conducted in the absence of any commercial or financial relationships that could be construed as a potential conflict of interest.

## Publisher’s Note

All claims expressed in this article are solely those of the authors and do not necessarily represent those of their affiliated organizations, or those of the publisher, the editors and the reviewers. Any product that may be evaluated in this article, or claim that may be made by its manufacturer, is not guaranteed or endorsed by the publisher.
