# Supplementary material for: Prognostic Role of Lymphocyte-C-Reactive Protein Ratio in Colorectal Cancer: A Systematic Review and Meta Analysis
Source: Front Oncol. 2022 Jul 6;12:905144. doi: 10.3389/fonc.2022.905144 (PMC9296779; doi:10.3389/fonc.2022.905144)
Supplement: Supplementary file 1 [file Table_1.docx]

Search Strategy

**PUBMED：**

#1 (((((((Colorectal Neoplasms) OR (Colorectal Neoplasm)) OR (Colorectal Tumors)) OR (Colorectal Tumor)) OR (Colorectal Cancer)) OR (Colorectal Carcinoma)) OR (Colorectal Carcinomas))

#2 (((lymphocyte c-reactive protein ratio) OR (lymphocyte/c-reactive protein ratio)) OR (LCR))

#3 #1AND#2

**EMBASE：**

No. Query Results Results Date

#14. #8 AND #13 58 6 Jan 2022

#13. #9 OR #10 OR #11 OR #12 3,685 6 Jan 2022

#12. 'lcr':ab,ti 3,675 6 Jan 2022

#11. 'lymphocyte/c-reactive protein ratio':ab,ti 20 6 Jan 2022

#10. 'lymphocyte to c-reactive protein ratio':ab,ti 26 6 Jan 2022

#9. 'lymphocyte c-reactive protein ratio':ab,ti 20 6 Jan 2022

#8. #1 OR #2 OR #3 OR #4 OR #5 OR #6 OR #7 212,897 6 Jan 2022

#7. 'colorectal carcinomas':ab,ti 5,340 6 Jan 2022

#6. 'colorectal carcinoma':ab,ti 19,620 6 Jan 2022

#5. 'colorectal tumor':ab,ti 3,010 6 Jan 2022

#4. 'colorectal tumors':ab,ti 4,951 6 Jan 2022

#3. 'colorectal neoplasm':ab,ti 785 6 Jan 2022

#2. 'colorectal neoplasms':ab,ti 1,960 6 Jan 2022

#1. 'colorectal cancer'/exp 202,435 6 Jan 2022

**Web of science**

#1 TS=(Colorectal Neoplasm* OR Colorectal Tumor* OR Colorectal Cancer OR Colorectal Carcinoma*)

#2 TS=(lymphocyte c-reactive protein ratio OR lymphocyte to c-reactive protein ratio OR lymphocyte/c-reactive protein ratio OR LCR )
